# Patent Medicines

**Published:** 1856-07

**Authors:** 


*Patent Medicines.*

We ask attention to the following, which first appeared in the *New York Journal of Medicine and Collateral Sciences.* It shows very clearly that probably four-fifths of the medicines and nostrums, sold under the pretended protection and importance of a patent or copyright, are entirely destitute of any such legal protection and fraudulent. It is a subject on which both the profession and the community should be better informed.

"*The Sale of Patent Medicines a Violation of Law—Important Communication from the Clerk of the District Court of the Northern District of New York.*

"The following correspondence, which we now for the first time make public, clearly establishes the fact, that the traffic in patent medicines (one of the greatest curses from which the community suffers, by the imposition of empirics,) is done under the most shallow pretence of legality. It has always been a mystery to us how honest men could be so heedless of public good, as to legalize the indiscriminate sale of such dangerous compounds as many of these nostrums are; but we here have explained the whole secret. As might have been anticipated, from the character of the men engaged in their manufacture, the sale of these drugs is effected fraudulently.

"We trust some measure will be taken to put a stop to the sale of these vile compounds, and bring to justice the violators of law, who poison public health and grow rich thereby. As these nostrums are not truly patented, the secret of their preparation being studiously withheld in defiance of the explicit declaration of our Patent Laws, we would suggest the passage of a law imposing a heavy penalty for the sale of 'patented' medicines, when in fact they are not patented, and allow half the fine recovered to the informer. It would be a truly philanthropic effort, worthy a Howard, or a Mrs. Fry, to secure the community from this terrible infliction of the sale and use of 'patent' medicines.

"We cannot sufficiently express our admiration of Mr. Conkling's integrity and enlightened discharge of his official duties. For several years he has uniformly refused to grant these applications to patent labels, from a conscientious belief in the illegality of such proceedings, and a conviction of the worthless character of such compounds. During this time he has not only sacrificed the income which is due from such grants, but incurred the odium and bitter hatred of this miserable class of impostors. In behalf of the medical profession we tender our acknowledgements for the service he has thus rendered directly to the community at large, and indirectly to legitimate medicine.

"From the annexed 'circular,' it will be seen that Mr. Conkling's views of the Patent Laws are endorsed by the State Department, and instructions in conformity therewith have been promptly issued by Mr. Secretary Marcy.—*Eds. New York Journal of Medicine.*"

"Buffalo, March, 1856.

"*Dear Sir*—Will you do me the personal favor to furnish me with a written statement of your views on the subject of copyrights for labels, etc., as expressed to me in a conversation yesterday.

"Coming from a gentleman of your rank and position, I am certain that they would possess interest; and I would like to give them publicity through some of our medical journals.

"Sincerely wishing that the public had many more such faithful servants, I remain yours truly,

FRANK H. HAMILTON.

AURELIAN CONKLING, Esq."

"CLERK'S OFFICE, *Dist. Court of the U. S., for the Northern District of New York.*

BUFFALO, March 18, 1856.

"*Dear Sir*—I am much obliged for the interest manifested by you in the subject with which I lately troubled you; and although it is probable that in your politeness you over-estimate the importance of the views expressed to you, and of those contained in my letter to the Hon. S. G. Haven, still it is possible that they may be useful to others, and I therefore proceed to repeat them here, for such use as you may deem expedient. I am much pleased with the polite interest you manifest in the subject under consideration, because I regret to say I have heretofore experienced very different treatment from some persons who have found their way into your profession, and who not only so far forgot their professional obligations as to manufacture nostrums, but who are also guilty of low abuse of an officer whose sense of duty would not admit of his being made instrumental, improperly, in imposing their mixtures upon the public.

"The immediate purpose of my letter to our representative in Congress, was to invoke the attention of the Department of State to what, it seems to me, is a great abuse and perversion of the provisions of law in relation to *copyrights*. The subject of copyrights is under the general supervision and control of the State Department of the United States; and officers who have subordinate duties to perform must, to some extent, be subject to directions and instructions from that department. Applications are frequently made to me to record, under the provisions of law above alluded to, labels of medicines, compounds, and mixtures, of different grades of pretension, from an 'elixir of life,' or a 'diarrhœa cordial,' to a hair-dye or a corn-salve.

"Upon the occurrence of the first application of this sort, a number of years ago, being asked my reasons for refusing to treat the subject as one embraced in the provisions of the Act, entitled 'An Act to amend the several Acts respecting copy rights,' I made the following reply, a recital in part of which will express the views which have ever since governed me upon the subject.

" 'I am sorry that my views of the Act of Congress, abovementioned, are such as to interfere with your interest or wishes.

'It is not the province of Clerk of the District Court to *grant* anything. His duties are ministerial, and upon the subject of copyrights he is bound to do what he is directed to do by the Act of Congress.

'If I should record the *label* sent by you, and should send you a certificate of the fact, I would thereby *grant* you nothing, nor would you gain anything, unless the Act of Congress embraces such a subject.

'I have examined with considerable care the Act of Congress abovementioned, and I will state some of my views upon it, from which you will infer that I do not think proper to record a *label* under that Act.

'My opinion is, that the *map*, *chart*, *musical composition*, *print*, *cut*, *or engraving*, must have a value as such, and be intended for sale as such; that whichever it may be, print, cut, or engraving, *it* must have a *title* applicable to itself, which is to be recorded.

'I am also of opinion that the Act of Congress was designed to promote the acquisition and diffusion of knowledge, and to encourage the production and publication of works of art, the general purpose being to advance the people in civilization and refinement.

'I think, furthermore, that, by the Act to which I have referred, Congress did not intend to prevent the imitations of the stamps and labels of any manufactured article, or goods, or merchandise. That is a subject of such extensive interest and importance, that, if it had been the intention of Congress to embrace it in the provisions of the law, that intention would have been distinctly and unequivocally manifested. It is not likely, however, that such a provision by Congress will ever be found so much out of place as it would be in *An Act to amend the several Acts respecting* COPYRIGHTS.

'Furthermore, I am quite certain that Congress did not intend that this Act should be so prostituted as to be made instrumental in deluding the ignorant and inconsiderate into the purchase and use of the various nostrums, catholicons, and panaceas, which are so much worse than useless to the community.'

"It is the practice, I am informed, in many of the Judicial Districts, to make records of such labels as are abovementioned, and I suppose it is done to avoid the trouble and ill-will engendered by a refusal. It is entirely clear, however, that such a practice is not in accordance with the intention and design of the Act of Congress abovementioned; and I have no doubt that if the mischiefs of the practice were realized, it would be discontinued.

"The course of proceeding abovementioned, is that by which almost the whole number of the miscalled 'patent medicines' are brought forth. It would seem to be unnecessary to state, that there is no force or validity whatever in this proceeding for such pretended purpose.

"There are means provided in the Patent Laws for securing to any individual the exclusive right to 'any new and useful art, machine, manufacture, or composition of matter, or any new and useful improvement on any art, machine, manufacture, or composition of matter,' which he may invent: and the necessary requirements, in order to accomplish the purposes, are clearly and definitely prescribed as follows—'But before any inventor shall receive a patent for any such new invention or discovery, he shall deliver a written description of his invention or discovery, and of the manner and process of making, constructing, using, and compounding the same, in such full, clear, and exact terms, avoiding unnecessary prolixity, as to enable a person skilled in the art or science to which it pertains, or with which it is most clearly connected, to make, construct, and use the same.'

"The Act also provides, that the inventor shall accompany his application 'with specimens of ingredients, and of the composition of matter, sufficient in quantity for the purpose of experiment, where invention or discovery is of composition of matter.' The Act also provides, that the applicant shall make oath, that he does not know or believe that the composition of matter was ever before known or used. These, and the other requirements of the law being complied with, provision is made for a critical examination into the merits and character of the alleged invention; and 'if the commissioner shall deem it to be sufficiently useful and important, it shall be his duty to issue a patent therefor.' This, it will be perceived, is a different course of procedure from that of filing a label, not even indicative of the character of the compound it is to cover. The law, it will be perceived, provides for a truthful statement of the ingredients and proportions of every patented compound. The purposes and effect of the provision are twofold. In the first place, the means are afforded for an intelligent and careful examination into the compound, in order to determine whether it is worthy of the countenance of the Government; and, secondly, after the termination of the duration of the privileges secured by the letters patent, the necessary knowledge is at hand to make the invention directly available to the public, by furnishing to all a knowledge of its ingredients and mode of preparation. Moreover, the 'letters patent' themselves, in accordance with their true purport, contain a plain statement of these particulars. There is something *open* in these requirements, and in the whole course of proceeding marked out in relation to patents; and the fact is, just as one would suppose, that there are really very few 'patent medicines.' Those medicines, sold as such, are nearly all of them utterly destitute of any real basis for the pretence under which they are imposed upon the public.

"If the practice of recording labels of medicines shall be discontinued in the clerk's offices, one important step will be taken towards clearing away the delusion which prevails upon the subject. And if your profession, with that true regard for the general public good which characterizes its *worthy* members, will take the subject in hand, I have no doubt that you can obtain the enactment of penalties against the sale of any medicines under the pretence that they are, when in truth they are not, patent medicines. There has been legislation to prevent the adulteration of medicines; but it seems to me, that it is a more important end to shield the people against the miserable mixtures which, as things are now managed, are, by the apparent encouragement of the Government, imposed upon them. I regret, dear Sir, that this letter has necessarily been so hasty; I do not mean, however, to intimate that its positions are not deliberately taken. Very respectfully,

Your obedient servant,

DR. FRANK H. HAMILTON." AURELIAN CONKLING.

---

"CIRCULAR.

DEPARTMENT OF STATE,

WASHINGTON, April 11, 1856.

*Clerk of the District Court of the United States.*

"*Sir*—The Act of Congress, approved February 3, 1831, entitled, 'An Act to amend the several Acts respecting copy-rights,' is 'An Act for the *encouragement of learning*, by securing the copies of maps, charts, and books, etc., to the authors and proprietors of such copies;' and inasmuch as mere *labels* are not comprehended within the meaning of said Act, you will for the future refuse, in all cases, to record or issue a certificate for the same under said Act. I am, Sir, very respectfully,

Your obedient servant,

W. L. MARCY."

